# Endometriosis: A Disease That Remains Enigmatic

**DOI:** 10.1155/2013/242149

**Published:** 2013-07-17

**Authors:** Pedro Acién, Irene Velasco

**Affiliations:** ^1^Department of Obstetrics and Gynecology, San Juan University Hospital, 03550 Alicante, Spain; ^2^Department/Division of Gynecology, School of Medicine, Miguel Hernandez University, Campus of San Juan, 03550 Alicante, Spain; ^3^Instituto de Ginecología P.A.A., 03002 Alicante, Spain; ^4^Departamento/Area de Ginecología, Facultad de Medicina de la Universidad “Miguel Hernández,” Campus de San Juan, 03550 Alicante, Spain

## Abstract

Endometriosis, a gynecologic pathology, is defined by the presence of a tissue similar to uterine endometrium, which is located in places other than physiologically appropriate. These endometrial heterotopic islets contain glands and stroma and are functionally capable of responding to exogenous, endogenous, or local hormonal stimuli. Endometriosis affects 8%–10% of women of reproductive age; in 30% of the women, the condition is associated with primary or secondary infertility. In several instances, endometriosis persists as a minimal or mild disease, or it can resolve on its own. Other cases of endometriosis show severe symptomatology that ends when menopause occurs. Endometriosis can, however, reactivate in several postmenopausal women when iatrogenic or endogenous hormones are present. Endometriosis is occasionally accompanied by malignant ovarian tumors, especially endometrioid and clear cell carcinomas. Its pathogenesis is widely debated, and its variable morphology appears to represent a continuum of individual presentations and progressions. Endometriosis has no pathognomonic signs or symptoms; it is therefore difficult to diagnose. Because of its enigmatic etiopathogenesis, there is currently no satisfactory therapy for all patients with endometriosis. Treatments include medications, surgery, or combined therapies; currently, the only procedures that seem to cure endometriosis are hysterectomy and bilateral salpingo-oophorectomy. In this paper, we review the most controversial and enigmatic aspects of this disease.

## 1. Introduction

Endometriosis is a gynecologic pathology that is frequently considered enigmatic; it is defined by the presence of a tissue similar to uterine endometrium that is located in places other than physiologically appropriate (i.e., uterine endometrial cavity), most commonly in the pelvic cavity, including the ovaries, the uterosacral ligaments, and the pouch of Douglas. These endometrial heterotopic islets contain glands and stroma and are functionally capable of responding to exogenous, endogenous, or local hormonal stimuli.

Despite its first description as a pathology three centuries ago and recognition as a clinical entity by Sampson since 1918–1920 [1], the issue of the proper characterization of endometriosis as a disease, a clinical entity or a pathology is still a topic of discussion today. However, endometriosis affects 8%–10% of women of reproductive age; in 30% of these women, endometriosis is associated with primary or secondary infertility [2, 3]. The presentation and evolution of the disease are variable; in some cases, the disease can persist as a minimal or mild disease, or the disease can also disappear. Other cases can show severe symptomatology because of invasion and tissue infiltration, growth of endometriomas or “chocolate cysts,” severe pelvic adhesions, or pelvic blockage that can affect other organs. Endometriosis usually ends when menopause occurs because of the decrease in estrogen level during menopause. Endometriosis can, however, reactivate in some postmenopausal women when iatrogenic or endogenous hormones are present [4–6].

Although the disease is recognized as benign, endometriosis is occasionally accompanied by malignant ovarian tumors, especially endometrioid and clear cell adenocarcinomas. Its own identity in some patients appears to be malignant because of its level of progression, the affected organs, and the recurrence of the disease. Therefore, the pathogenesis and physiopathology of endometriosis remain widely debated, and its variable morphology characterizes its biology or natural history not as a single entity but instead as a continuum of individual presentations and progressions [2, 7–10].

Endometriosis has no pathognomonic signs or symptoms and is therefore difficult to diagnose. Similarly, there is currently no satisfactory therapy for all patients with endometriosis. Currently, the therapies for endometriosis are controversial because they can improve pain and infertility but they do not cure the disease. Hysterectomy with double adnexectomy is the only surgical method that eliminates the disease, but it is undesired or contraindicated in young patients or women who may someday wish to become pregnant. Conservative surgery via laparotomy or laparoscope is usually used to manage these patients and is often combined with hormonal treatment, which is also controversial.

The only certainty about endometriosis is that it is a disease in women and in some menstruating primates. Emery Wilson stated that endometriosis “remains a riddle wrapped in a mystery inside an enigma” [2].

## 2. Historical Data

Although endometriosis is considered a disease of the 20th or 21st century, the first descriptions of endometriosis are ancient. The first references to endometriosis-associated symptoms are found in the Ebers Papyrus (Tebas, Egypt, 1500 B.C.), in which a treatment for a “painful disorder of menstruation” is described. However, a more detailed description of peritoneal endometriosis was made in Daniel Shroen's 1690 book titled “Disputatio Inauguralis Medica de Ulceribus Ulceri,” in which he referred to adhesions and endometriomas as complications associated with the disease. Knapp performed an interesting historical review of endometriosis in the 17th and 18th centuries [11]. In the 18th century, scientists from England, Germany, Holland, and Scotland described endometriosis in autopsy studies and reported important descriptive facets: it is a disease in women (A. Ludgers: Dissertatio medico-practica inauguralis de hysterilide, Lovain, 1776); it appears after the first menstruation (S.C. Duff: Disertatio Inauguralis medica de metritide, Louvain, 1769; C. Stolzel: Demetrifides diagnosi et cura, Leipzig, 1797); and it is associated with the uterine area and especially with pelvic pain, infertility, and recurrent miscarriages (J. Gebhard: Dissertalia medica de inflamatione uteri, Marburg, 1786). The medical literature in the 19th century referred to cyst-like lesions associated with endometriosis.

Carl von Rokitansky in 1860 provided the first identification and detailed description of endometriosis. The term “chocolate cyst” was used for the first time by Breus in 1894. Von Recklinghausen, Cullen, O'Frankl, and others [12–14] later studied endometriosis. In 1896, Cullen called attention to glandular inclusions derived from the mucous membrane of the uterus. In 1903, Runge described in detail the endometriomas, and R. Meyer described endometriosis in an abdominal scar and later described intestinal endometriosis. Bljair Bell de Liverpool used the terms “endometriosis” and “endometriomas,” which Sampson also used years later. Several hypotheses regarding the pathogenesis of endometriosis were proposed during this period. In 1905, Pick suggested the persistence of Wolffian rests; in 1924, Halban proposed lymphatic dissemination as the origin of endometriosis.

Serving as a critical reference for endometriosis today is a paper published by Sampson in 1921 [15], which was titled “Perforating Hemorrhagic (Chocolate) Cysts of the Ovary.” This document is especially important because it relates to pelvic adenomas of endometrial type (“adenomyoma” of the uterus, rectovaginal septum, sigmoid, etc.). This study documents, in great detail and with interesting drawings, the pathologic findings of 23 cases of hemorrhagic (chocolate) cysts that perforated the ovary (endometriomas). By operating on two patients at the time of their menstruation, Sampson found that the cysts were lined with a tissue similar to endometrium, which demonstrated evidence of menstrual shedding and was therefore an ectopic tissue that was functionally similar to endometrium; therefore, he called the disease “endometrial adenomas.” In 1922, Sampson [16] published another key work on the surgical treatment of the endometrial intestinal adenoma. In 1927 [17], he formulated a new concept in the article titled “Peritoneal Endometriosis due to the Menstrual Dissemination of Endometrial Tissue into the Peritoneal Cavity.” The hypotheses for the origin of endometriosis from his 1927 article dominated the criteria and the scientific literature on endometriosis for the next 80 years. John Albertson Sampson (1873–1946) from Albany, New York, worked only in a private practice and published >20 articles from 1921 to 1940. He established the basis for considering endometriosis as a clinical entity; he was the first to suggest the retrograde menstruation and implantation theory as its origin. Sampson proposed a surgical treatment for endometriosis and described the different lesions that can occur in the disease (chocolate cysts, adhesions, adenomyomatosis, rectovaginal septum nodules, and deep infiltrations). Sampson also described the relationship of the disease with malignant ovarian tumors.

The number of publications dedicated to endometriosis increased after 1921, which was especially due to discussions regarding its histogenesis (Cullen, Meyer, and Sampson), although the word “endometriosis” did not yet appear in the book indices. We reviewed several books of the late 19th and early 20th centuries and only found a broad description of endometriosis and its complications in the book “Ginecología Operatoria” by H. S. Crossen and R. J. Crossen from 1940 [18].

Over the last 30 years, numerous publications addressing this complex disease have focused on endometriosis-associated infertility and the different etiopathogenic and pathophysiological mechanisms involved in its enigmatic biology, as well as the design of new medical therapies.

## 3. The Importance of the Problem

Endometriosis remains a controversial disease despite (1) being a well-known condition that affects a large number of women; (2) current scientific and technological advances, numerous lines of research and investigators addressing the disease; and (3) the existence of a journal and various regular international congresses dedicated specifically to the disease.

Endometriosis is a major problem for women, health systems, and society. Over the last 30 years in our clinic, more than 6% of the women seen in a general gynecological consultation for pathology, contraception, or a checkup had endometriosis. Given that these patients often returned multiple times for gynecological assistance, over 20%–30% of the gynecological consultations are likely related to endometriosis. In the last year, 17% of the surgeries in our department (including laparoscopies, laparotomies, vaginal hysterectomies, and breast cancer) showed evidence of endometriosis. Excluding the patients with endometriosis who required surgery, most of our patients likely had minimal or mild endometriosis, and only 3%-4% of the women had moderate-to-severe endometriosis. These findings involve a significant number of patients.

To estimate the socioeconomic burden of the illness caused by surgically confirmed endometriosis in Canada in 2009, including direct health care costs, lost productivity, and lost leisure time costs, Levy et al. [19] conducted a study in clinics in Alberta and Quebec. The estimated mean annual societal cost of endometriosis was $5,200 per patient (95% CI, $3,700 to $7,100), with lost productivity and lost leisure time costs accounting for 78%. Extrapolating these figures yields an estimated total annual cost of $1.8 billion (95% CI, $1.3 billion to $2.4 billion) attributable to surgically confirmed endometriosis in Canada.

Similarly, Oppelt et al. [20] estimated the financial burden of inpatient costs for endometriosis treatment in Germany in 2006. A total of 20,835 patients were admitted to the hospital for endometriosis treatment (1.27 per 1,000 women of reproductive age). The average cost per patient was estimated at 3,056.21€. The total inpatient costs for endometriosis treatment in 2006 were estimated at 40,708,716€. The surgical procedure most often performed in treating endometriosis was hysterectomy (24.70% of cases). Prast et al. [21] performed this same cost analysis in Austria. The average annual costs of one case of endometriosis are 7,712€, with 5,605€ attributable to direct costs and 2,106€ to indirect costs. This financial report indicates an overall economic burden of 328 million €. Inpatient care (45%) and loss of productivity (27%) were identified as the major cost factors. The patients themselves pay for 13% of the medical costs (out-of-pocket expenses). The overall economic burden of endometriosis in Austria is currently comparable to that of Parkinson's disease [21]. 

Greater medical expenses were observed in Belgica in 2008. Simoens et al. [22], in the data analysis of 909 women, demonstrated that the average annual total cost per woman was 9,579€ (95% CI, 8,559–10,599€). Productivity loss of 6,298€ per woman was double the health care costs of 3,113€ per woman. Health care costs were primarily attributable to surgery (29%), monitoring tests (19%), hospitalization (18%), and physician visits (16%). Endometriosis-associated symptoms generated 0.809 quality-adjusted life years per woman. Decreased quality of life was the most important predictor of direct health care and total costs. Costs were greater with increasing severity of endometriosis, presence of pelvic pain, presence of infertility, and a greater number of years from the time of diagnosis. Considering the importance of this issue, Simoens et al. [23] proposed the EndoCost study, which allows a cost-of-illness analysis to justify the prioritization of future research in endometriosis.

Surgical laparoscopy with intestinal resection is a frequently performed practice today. The procedure is described in a large number of publications as a treatment for deep-infiltrating endometriosis (DIE). Technological advances and diagnoses have contributed to an increased use of this procedure in recent years. However, personal suffering and the cost of the procedure should be weighed when performing it, especially when the procedure is performed on young women. Finally, when used routinely, the laparoscopic diagnosis can be costly; our group only performs this type of surgery in women who have minimal or mild endometriosis when it is necessary to diagnose (and treat) a possible disease-associated infertility.

From this point forward, we mention the more relevant and controversial aspects of endometriosis and possible future therapies for this disease.

## 4. Types, Location, and Morphology of Endometriosis

There are two classical, well-differentiated endometriosis entities in both clinical manifestations and etiopathogenesis: (1) adenomyosis or internal endometriosis and (2) external endometriosis or simply endometriosis. (1) Adenomyosis or internal endometriosis occurs when ectopic endometrial foci infiltrate the outer muscular walls of the uterus. Adenomyoma is an area of adenomyosis that is encapsulated in the myometrial tissue. We know today that some of these masses can be another type of pathology (ACUM, [24, 25]). (2) External endometriosis or simply endometriosis is present when the endometrial ectopic foci are located anywhere within the pelvis (ovary, pouch of Douglas, uterosacral ligaments, rectovaginal septum, and vesicouterine pouch), abdominal cavity (bowel, omentum), or outside (lungs, brain). However, many authors [26, 27] refer to deep endometriosis as “adenomyosis,” that is, those foci located in the rectovaginal septum or infiltrating the pouch of Douglas or the bowel (DIE). These nodules, composed of glands and scarce stroma, are surrounded by hyperplastic smooth muscle cells and cause severe clinical symptoms.

Regarding location, the endometriotic implants have been found almost anywhere in the female body, but they occur more frequently in the pelvic cavity. The most commonly affected areas are the ovaries followed by the pouch of Douglas, uterosacral ligament (especially its insertion into the back side of the uterus), vesicouterine pouch, serosal surface of the uterus, fallopian tubes, round ligament, and rectovaginal septum (which is along the ovaries, the most common site of recurrence or malignancy). Endometriosis can also be located inside the genital tract and can spread into the cervix and vagina, especially the posterior vaginal wall, which is related to the frequently affected rectovaginal septum. Endometriotic implants can be found in the perineum (especially on scars of previous episiotomies) or in the Bartholin gland. Other extragenital locations for endometriosis are the gastrointestinal tract (appendix, rectum, and sigmoids), the urinary tract, or the thoracic area.

### 4.1. Macroscopic Morphology

Endometriosis presents a wide range of macroscopic variability; visual assessment (during laparoscopy) of an operative situs with minimal and mild endometriosis is subject to a considerable interindividual variability [28]. In more advanced cases, it is the same. In general, one or both ovaries are cystic, thickened, and adhere to the posterior side of the uterus and to the broad and uterosacral ligaments. The size of endometriomas can range from 1 cm to 6 cm but can also reach 14-15 cm. These cysts break frequently, and their characteristic chocolate fluid can reach the abdominal cavity. This chocolate fluid contains elevated amounts of fibrinogen and iron that generate strong, dense adhesions to nearby organs and can precipitate acute abdominal pain that requires urgent surgical intervention. The fallopian tubes are usually free of endometriosis, but they are sometimes affected by the pelvic adhesions characteristic of endometriosis. Adhesions can be firm and rough bands of fibrous tissue with hemorrhagic areas. This type of infiltrating endometriosis is primarily located in the uterosacral region, the rectum or sigmoid anterior walls, the ovaries, and the posterior side of the uterus. Endometriosis that block the pouch of Douglas can cause complex and difficult interventions. In external locations, such as a laparotomic scar or the perineum or umbilicus, endometriosis can appear as a painful, hard, blue-black, or brown nodule that grows during menstruation. In the cervix, endometriosis may be present as velvety red lesions during menstrual cycles that may darken to a deep purple color during bleeding.

### 4.2. Microscopic Aspects

Endometriosis is microscopically diagnosed as the presence of glands and stroma. This ectopic tissue may present cyclical changes in which the glands show a minimal proliferative activity or an inadequate secretory transformation. This activity occurs because endometriotic lesions express estrogen and progesterone-specific receptors [29] with a distribution similar to eutopic endometrium, although in a lower concentration and without expression of the progesterone B-receptors. Its response to hormonal stimulation is therefore variable. The ectopic endometrium does not often change during the menstrual cycle due to the strong inflammatory reaction that is triggered proximal to it. This inflammatory reaction causes a dense atrophic scar that may affect blood flow toward the endometriotic focus, thus decreasing its response to hormonal changes. Other molecular alterations in endometriosis, such as the aberrant expression of the active P450 aromatase enzyme [30] and its stimulation by IL-6 or TNF-*α* [31, 32], lead to a continuous local supply of estrogen independent of circulating levels.

In histological samples, a third of the typical clinical endometriosis cases show no endometrial tissue layer but a large number of leukocytes, histiocytes, and hemosiderin-containing macrophages (or siderophages) in an important connective component. This lesion is also recognized as endometriosis and is a consequence of repeated menstrual desquamations and pressure on the lesion due to blood retention in the cystic cavity.

Stroma vessels may contain thrombi that cause an infarcted area and therefore a self-destruction of the endometriotic lesion. Consequently, the remaining cells may show pyknotic nuclei similar to atypical endometriosis. These pyknotic nuclei can actually be atypical endometriosis with a p53 protein overexpression. Likewise, a squamous metaplasia or a uterine tube-like epithelium (endosalpingiosis) may be found in other cases.

## 5. Histogenesis

During the first part of the 20th century, several theories regarding the histogenesis of endometriosis were proposed based on clinical and experimental evidence. The theories were reviewed by Ridley [33] and grouped into three categories (1) transplantation, (2) coelomic metaplasia, and (3) metaplasia induced by factors released into the peritoneal cavity. Therefore, there are actually two theories (transplantation and metaplasia) to which hypotheses of ectopic Müllerian rests must be added. Sampson's transplantation and implantation theory (also called the theory of retrograde menstruation) is the most widely accepted theory for the formation of ectopic endometrium in endometriosis because it may explain nearly all cases. This theory suggests that the origin of endometriosis is due to the propagation and attachment of eutopic endometrial cells (endometrium) outside the uterus, in a continuous manner (adenomyosis), through the fallopian tubes (pelvic endometriosis), or by lymphatic, hematogenous or mechanical dissemination (perineum or laparotomic scars).

These theories (metaplasia, Müllerian rests, and retrograde menstruation) appear to be contradictory. According to the first two hypotheses, endometriosis originates in the peritoneal serosa or structures derived from the Müllerian ducts, whereas according to the retrograde menstruation theory, its origin is due to transplantation and implantation of uterine endometrial cells. However, the development of metaplasia or growth of Müllerian rests may occur due to menstrual debris that reach the pelvic peritoneum through retrograde menstruation. Thus, these theories could be considered complementary.

Both endometrial implantation and coelomic metaplasia can give rise to minimal or microscopic peritoneal lesions (papillary to red lesions), but they are so frequent that they should not be considered a disease but rather a physiological endometriosis [34]. These implants usually resolve spontaneously or progress to mature “black” lesions and white scar lesions [35]. In certain women, other implants grow and develop into “endometriotic disease,” which is characterized by the presence of dense adhesions (affecting local physiology), endometriotic ovarian cysts and DIE (rectovaginal septum nodules).

Nisolle and Donnez [35] proposed three types of endometriotic lesions: peritoneal, ovarian, and rectovaginal. After analyzing these lesions, they proposed a different theory of histopathogenesis: retrograde menstruation for peritoneal endometriosis, mesodermal Müllerian rests for rectovaginal endometriotic nodules, and metaplastic histogenesis for ovarian endometriotic lesions. However, retrograde menstruation is the most widely accepted theory. Phagocytosis or apoptosis resolves peritoneal implantation in more than 90% of the cases (physiological endometriosis). However, these implants can grow and progress to cause “endometriotic disease” only in certain women with altered or deficient immune systems.

The most controversial aspects in endometriosis are related to its physiopathology and pathogenesis.

## 6. Evolutive Biology: Etiopathogenesis and Physiopathology

Although it has been established that endometriosis occurs only in certain women, there are factors that increase the risk for developing the disease, such as age and genetic and environmental factors, as well as the interactions between these factors [36]. There is a genetic and constitutional predisposition with inherited tendency, as pointed out by Simpson et al. [37] who referred to a polygenic/multifactorial inheritance. Twin and family studies have documented an increased relative risk in families. There appears to be a 7% recurrence risk for all first-degree relatives of someone with endometriosis. Having severe endometriosis is more likely when a relative has the disease (61%) than for women without an affected first-degree relative (24%) [37, 38]. The disease is more common in Caucasians than in other ethnic groups, but no association with HLA antigen distribution has been found [39].

### 6.1. Genetic Factors

To identify the genetic factors that contribute to endometriosis, two genome-wide association studies (GWAS) have been conducted in two different ethnic populations but the results must still be replicated consistently and across various ethnicities. Previous studies have found genetic associations with endometriosis for single-nucleotide polymorphisms (SNPs) at different chromosome loci in Caucasian and Japanese populations [40–42]. GWAS have identified a locus at 7p15.2 associated with endometriosis in the study by Painter et al. [41]. The results from Falconer et al. [43], who studied GWAS and transcriptome sequencing, have demonstrated that genes located in the 1p36 region are important in both endometriosis and endometriosis-associated cancer development. Recently, WNT4, CDKN2BAS, and FN1 have been confirmed as the first identified common loci for endometriosis in the Caucasian population [44]. Therefore, GWAS demonstrate that some genes are involved in endometriosis and ovarian carcinoma, but all of this information is in the initial research phase.

Second, Wang et al. [45] studied the circulating microRNAs identified in a genome-wide serum microRNA expression analysis as noninvasive biomarkers for endometriosis. They demonstrated that the circulating miRNAs miR-199a, miR-122, miR-145, and miR542-3p could potentially serve as noninvasive biomarkers for endometriosis. Furthermore, miR-199a may also play an important role in the progression of the disease. Other proteomic analysis and plasma microRNA expression patterns are currently widely investigated as novel biomarkers for endometriosis and endometriosis-associated ovarian cancer [46, 47].

### 6.2. Lifestyle Factors and Endocrine Disruptors

Age is a risk factor for endometriosis (usually after 19-20 years of age); if endometriosis occurs before 19-20 years of age, an obstructive genital malformation should be ruled [48–51]. Other risk factors include race, socioeconomic status, height, and weight. Endometriosis occurs more frequently in taller and thinner women. Lifestyle, early menarche, having longer periods, obstetric history, and contraception are potential but doubtful factors [3, 52]. Likewise, women with naturally red hair may have an increased risk for developing endometriosis because of a possible association with altered coagulation and immune functions [53]. We have already mentioned these related factors: the familial predisposition, genital anomalies, and the association with infertility or recurrent pregnancy losses. There is evidence that with the presence of genetic susceptibility, retrograde menstruation, uterine, peritoneal, and environmental factors (such as dioxins) may lead to developing the disease [54–58].

Now being studied in depth is the influence of endocrine disruptors (bisphenol A, phthalates) on reproductive health and endometriosis development [59]. There is increasing concern about chemical pollutants that are able to mimic hormones, the so-called endocrine-disrupting compounds (EDCs), because of their structural similarity to endogenous hormones, their ability to interact with hormone transport proteins, or their potential to disrupt hormone metabolic pathways. Thus, the effects of endogenous hormones can be mimicked or, in some cases, completely blocked. A substantial number of environmental pollutants, such as polychlorinated biphenyls, dioxins, polycyclic aromatic hydrocarbons, phthalates, bisphenol A, pesticides, alkylphenols, and heavy metals have been shown to disrupt endocrine function [60]. Endocrine disruptors in utero cause ovarian damages linked to endometriosis [61]; and the prenatal exposure of mice to bisphenol A elicits an endometriosis-like phenotype in female offspring [62]. Caserta et al. [63] have also studied the influence of endocrine disruptors in infertile women. The percentage of patients with detectable bisphenol A (BPA) concentrations was significantly higher in the infertile patients compared to the fertile subjects. Patients with endometriosis had higher levels of peroxisome proliferator-activated receptor gamma (PPAR*γ*) than all women with other causes of infertility.

### 6.3. Inflammatory/Immune Factors

The primary issue with endometriosis is accepting the theory of Sampson and knowing the existence of retrograde menstruation in all women, why do only some women develop endometriosis? If retrograde menstruation is a physiological process that occurs in most of the women and only <8% to 10% of the women who develop the disease, other factors must determine its progression. In a revision [34], two suggestions for progression have been postulated: (1) there are intrinsic anomalies of eutopic endometrium that develop resistance from elimination by peritoneal immune cells and (2) the disease is a consequence of an altered function of peritoneal macrophages and natural killer (NK) cells that are unable to eliminate the endometriotic implants. The relationship between these theories is not clear; they are likely interdependent. The peritoneal environment may induce alterations in the ectopic endometrial tissue in those with a genetic predisposition, thus facilitating implantation and invasion. However, an excess of refluxed endometrium may induce a proinflammatory and hormonal environment that produces endometrial changes and favors the metaplasia of coelomic epithelium, which is already altered by peritoneal inflammation. Some molecular alterations described in endometriosis are related to disorders of angiogenesis and dysregulation in the apoptosis of immune and ectopic endometrial cells. Other authors have supported the autoimmune nature of endometriosis independent of its relationship with the factors mentioned previously, but it is likely that disease progression in these patients is accelerated by an immunological dysregulation or immunotolerance. Embryotoxicity reported in the serum and peritoneal fluid of infertile women with endometriosis appears to be related to high levels of IL-6, IL-8, and NK cells in these fluids [64].

It is accepted that most cases of minimal-to-mild endometriosis are physiological and temporal processes that resolve via the cytolysis of attached endometrial cells. The immune response and its consequences, which are triggered to eliminate these implants (unruptured luteinized follicle, hyperprolactinemia, alterations of tubal motility, and phagocytosis of gametes), as well as an altered peritoneal environment, could be responsible for endometriosis-associated infertility. The disease does not progress in immunocompetent women, and a temporary infertility that is similar to that in women with unexplained subclinical infertility occurs. However, in other cases, genetic or constitutional predisposition and immunotolerance to endometrial antigens (decreased NK activity and T-cell anergy) lead to the progression of endometriosis. This progression presents with infiltrating nodular and cystic lesions with obvious clinical manifestations that advance toward more severe stages. In these cases, infertility is caused by mechanical factors, such as adhesions, tubal distortion, or altered oocyte quality [65, 66].

To restore this immunological dysregulation, we developed several different clinical trials using intracystic recombinant IL-2 as an immunomodulatory agent in patients with endometriomas, but our results were inconclusive [67–69].

### 6.4. Stress and Local Steroid Modulation

Recently, the most interesting hypotheses regarding the etiopathogenesis of endometriosis are related to stress, inflammation, and local hormonal changes. Tariverdian et al. [70] suggested the concept of endometrial dissemination as a result of a neuroendocrine-immune disequilibrium in response to high levels of perceived stress caused by cardinal clinical symptoms of endometriosis. This stress induces a vicious cycle, aggravating peritoneal inflammation and angiogenesis, and, consequently, pain and infertility. The role of steroid hormones in the progression of endometriosis is also emphasized in this paper. Normal eutopic endometrium expresses the isoforms A (PR-A) and B (PR-B) of progesterone receptors; in the secretory phase, progesterone indirectly induces the 17*β*-hydroxysteroid dehydrogenase type 2 (17*β*-HSD-2), which converts estradiol (E2) to estrone (E1), leading to the apoptosis of endometrial cells. In ectopic endometrium, low levels of PR-A, no PR-B, and no 17*β*-HSD-2 are detectable. As a consequence, E2 accumulates and likely induces the proliferation of endometrial tissue. Moreover, the enzyme aromatase that is present in ectopic tissue creates E1, which is further converted to E2 by 17*β*-HSD type 1 (17*β*-HSD-1), thus contributing to the accumulation of E2. Additionally, E2 and proinflammatory cytokines upregulate the COX-2 expression that is overexpressed in endometriosis [71]. This activity stimulates cell division and angiogenesis and inhibits apoptosis; thus, the use of selective cyclooxygenase-2 inhibitors may suppress the growth of implants with an antiangiogenic effect.

Other alterations are related to the hormone dependence of ectopic endometrium, such as the expression of estrogen and progesterone-specific receptors [29] or the aberrant expression of active P450 aromatase [30]. This enzymatic activity gives rise to the conversion of circulating androstenedione into estrone in this tissue; this local estrogen production may promote the growth of endometriotic implants [72, 73]. We reported the presence of aromatase in the ectopic endometrium in 70% of the patients, whereas aromatase was absent in the eutopic endometrium [31]. This enzymatic activity may be regulated by a complex interaction of peritoneal macrophage-derived products as shown by its *in vitro* stimulation by IL-6, TNF-*α* or peritoneal fluid [31, 32], causing increased disease activity, disease severity and pelvic pain in these patients [74]. Therefore, aromatase inhibitors combined with GnRH analogues [75] or oral contraceptives [76] may be the next generation of endometriosis therapy.

## 7. Symptoms, Clinical Forms, and Staging

The most common symptoms of endometriosis are dysmenorrhea (during and at the end of menstruation), deep dyspareunia, chronic pelvic pain, and infertility in 30% of the cases. The intensity of symptoms may range from mild to severe, but the level of pain does not always relate to the seriousness of the disease.

The most frequent reason for consultation is persistent pain in one or both iliac fossae, often accentuated at the moment of ovulation and corresponding to the visualization of a hemorrhagic corpus luteum. Other patients attend emergency departments because of a sudden, acute abdominal pain during or after menstruation due to the rupture of endometrioma and the consequent peritoneal irritation. In an adequate medical interview, the patient usually notes other symptoms, such as premenstrual spotting for 2–4 days, headache, irritability, or premenstrual tension syndrome. However, because endometriosis symptoms do not always appear or may be caused by other conditions, its diagnosis cannot be based on symptoms alone. According to a different study, endometriosis is diagnosed using laparoscopy in a 4% to 52% range in patients with chronic pelvic pain. Therefore, although DIE correlates to pain, there is no clear explanation for the different symptoms of the disease. The possible causes are hemorrhagic corpus luteum, endometriotic cyst rupture, tissue infiltration, or perturbation of sensitive nerves [77].

In our opinion, there are three clinical forms of endometriosis.

1. Peritoneal endometriosis corresponds to minimal or mild endometriosis, and usually no progression is observed.
2. Cyst ovarian endometriosis is characterized by the presence of ovarian endometriomas that are unadhered or lightly adhered to the posterior side of the broad ligament.
3. Infiltrating and retracting endometriosis may present no evidence of endometriomas but may result in total pelvic blockage with uterus, bowel, and rectovaginal septum infiltration. This type of endometriosis is the most severe and disabling type. A deep adenomyotic infiltration of the posterior side of the uterus may be found using transvaginal ultrasound.

We have studied the correlation between these types of endometriosis and scaled their symptoms and examination and CA-125 values, and the results are inconclusive.

The scheme most widely used to classify the extent of the disease is the one employed by the American Society for Reproductive Medicine (ASRM), which was revised in 1996 [78, 79]. This staging system was established to predict fertility outcomes and does not correlate with the more common symptoms of pelvic pain. Unfortunately, the classification system is not adequately able to predict which women will suffer from infertility because infertility may occur in women with only mild endometriosis. In Dmowski's 1987 article [80], he compared the staging of endometriosis to the evaluation of the skin lesions to assign the severity of systemic lupus erythematosus. Certainly, moderate or severe cases are considered real instances of endometriosis.

## 8. Diagnosis

Endometriosis is diagnosed using medical interview, clinical examination, transvaginal ultrasound, and blood tests. We must consider two important signs: deep dyspareunia and nodules in the pouch of Douglas.

During the visual examination, we can observe certain forms of endometriosis in the vulva, perineum, laparotomic scar, or umbilicus. These lesions are dark blue nodules that are often dense and painful and increase in size during menstruation. Using the speculum, we may find similar nodules in the cervix or the posterior vaginal wall that are related to rectovaginal endometriosis. A nodule biopsy may be performed. Lesions, such as painful tumors fixed to the ovaries that create pelvic blockages, and dense and painful nodules in the uterosacral ligament, pouch of Douglas, and the retrocervical area, may be detected through a rectal or vaginal examination. The uterus may be retroverted, and its size may be increased by adenomyosis/adenomyomas or associated leiomyomas. The presence of galactorrhea or genital malformations must also be evaluated, especially in young patients.

Although endometriosis has no specific blood test, several screenings may be helpful in ruling out specific conditions in the differential diagnosis. A general biochemical analysis to discard other inflammatory processes or malignant tumors is necessary to perform. Although this analysis is usually normal in endometriosis, the doctor who evaluates the test results must evaluate certain values, such as sedimentation rate and tumor markers (especially CA-125), which are frequently elevated in these patients.

Novel biomarkers for endometriosis and endometriosis-associated ovarian cancer are currently under investigation [46]. Presently, there is no reliable noninvasive biomarker for the clinical diagnosis of endometriosis but circulating microRNAs (miRNAs) can serve as biomarkers [45]. Considerable effort has been invested in searching for less-invasive methods for diagnosing endometriosis. Previous studies have indicated altered levels of the CALD1 gene (encoding the protein caldesmon) in endometriosis. Meola et al. [81] have investigated whether average CALD1 expression and caldesmon protein levels are differentially altered in the endometrium and endometriotic lesions; they have evaluated the performance of the CALD1 gene and caldesmon protein as potential biomarkers for endometriosis. The presence of caldesmon in the endometrium of patients with and without endometriosis permitted diagnoses with 95% sensitivity (specificity 100%) and 100% sensitivity (specificity 100%) for the disease and for minimal-to-mild endometriosis in the proliferative phase of the menstrual cycle, respectively. In the secretory phase, minimal-to-mild endometriosis was detected with 90% sensitivity and 93.3% specificity. Therefore, caldesmon is a possible predictor for endometrial dysregulation in patients with endometriosis, but prospective studies are needed to confirm the potential of caldesmon as an exclusive biomarker for endometriosis [81].

We do not believe that an early diagnosis of endometriosis is essential. Although several authors have suggested that endometriosis may benefit from primary prevention measures [42], the reality is that if we admit that endometriosis does not currently have an effective treatment, invasive diagnostic efforts (e.g., laparoscopy) do not seem justified in cases of minimal or mild endometriosis with diagnostic purposes only. We have argued that its progression is debatable.

In practice, it is not easy to diagnose endometriosis due to its variability in symptomatology and its anatomic and clinical parallelism. Experience with the disease in a medical office assists in establishing a firm diagnosis more than what has traditionally been thought. Endometriomas are easily diagnosed using transvaginal ultrasound and blood tests. Deep endometriosis is often confirmed using a painful vaginal or rectal examination of nodules in the pouch of Douglas, rectovaginal septum, or uterosacral ligament. A useful tool to assess the severity of symptoms is a printed visual analogical scale for endometriosis, which includes the symptoms of dysmenorrhea, dyspareunia, and chronic pelvic pain and is graded on a 10-point scale [5, 67]. Thus, patients assess how they feel, and therapeutic results may be evaluated during the clinical follow-up.

Several examples of difficult diagnoses of endometriosis include the case of a patient who exhibited bilateral ovarian and DIE, adenomyosis, and intraluminal sigmoid endometriosis, the tumor of which was left in situ, after a previous laparotomy five years before showing only pelvic inflammatory disease. Another patient presented with recurrent hemorrhagic ascites and severe anemia. Surprisingly, ascites seemed to correspond to a large bleeding endometrioma. Similar cases are published in gastroenterology journals [82].

## 9. Treatments for Endometriosis

The etiology and pathophysiology of endometriosis have not yet been established. There is no treatment to permanently cure the disease. Treatments include hormonal medications (contraceptive pills, medroxyprogesterone, danazol, gestrinone, GnRH analogues, or levonorgestrel IUD), surgery (laparotomy or laparoscopy), or combined therapies. Medical treatments have shown to have limited effectiveness and can interfere with fertility during treatment and afterward. Hysterectomy with double adnexectomy is the only surgical method that eliminates the disease, but it is undesired or contraindicated in young patients or women who may wish to become pregnant. Conservative surgery via laparotomy or laparoscope is usually used to manage these patients and is often combined with hormonal treatment, which is also controversial.

The use of hormones is based on the evidence that the ectopic endometrium is modulated by sex hormones. Current hormonal management of endometriosis is based on two major mechanism of action: (1) iatrogenic menopause to create a hypoestrogenic hormonal climate to reduce the tropism of endometriotic lesions (GnRH agonists, GnRH antagonists, and aromatase inhibitor); or (2) pseudopregnancy to create a pseudodecidualization of the endometrium (progestins, intrauterine levonorgestrel-releasing system (LNG-IUD), intrauterine or vaginal danazol, and estrogen-progestin combination).

Current therapeutic approaches to endometriosis focus primarily on improving pain and reducing infertility. These approaches include the management of asymptomatic patients and medical or surgical interventions for symptomatic patients. Unfortunately, the surgical elimination or the medical suppression of endometriotic implants often provides only temporary relief because of the recurrent character of the disease unless radical surgery is performed. The treatment of choice depends on the potential for malignization and the patient's age, level of infertility, affected organs, severity of symptoms, or associated pelvic pathology.

Recent progress in the understanding of the pathogenesis of endometriosis has led to the development of new pharmaceutical agents that affect inflammation, immunomodulation, angiogenesis, or hormonal regulation. These new agents may prevent or inhibit the appearance of endometriosis or provide alternatives for noninvasive techniques for managing the disease. Several studies have suggested therapies based on immunomodulators, such as IL-2 [67–69], anti-TNF [83], or pentoxifylline [84]. The inhibitory enzymes aromatase [75, 76], COX-2 [73], or angiogenesis [85, 86] have also been proposed as effective therapeutic agents for endometriosis. Our group is currently developing a clinical trial for patients with endometriosis who receive oral aromatase inhibitors (anastrozole) with LNG-IUD in place. The preliminary results are not conclusive.

Regarding surgical treatment, several aggressive interventions are likely unnecessary. Despite the high morbidity rate, surgical excision of deep infiltrating bowel endometriosis (disc and segmental bowel resection) has become a popular treatment modality, particularly because operative laparoscopy techniques have improved. An increasing number of studies have reported numerous cases in which laparoscopic segmentary bowel resection has been performed, but the clinical reason or indication is often poorly documented. Several authors of these studies analyzed the quality of life for patients who underwent a laparoscopic colorectal resection [87] without comparing these patients to others who did not undergo a bowel resection. Although the quality of life improved for the majority of the patients who were treated with a colorectal resection, it is unclear whether a greater or similar health improvement could be achieved using a less aggressive surgery, with medical treatments only or with both medical treatments and a less aggressive surgery [88]. Therefore, surgery for the treatment of endometriosis that includes a bowel resection is rarely justified [89]. In our opinion, for many patients with a rectovaginal septum, endometriotic involvement can be managed without excising these lesions, and the affected patients may experience improvement merely by taking a low-dose contraceptive pill, an antiprostaglandin and an aromatase inhibitor. Additionally, a hysterectomy and bilateral salpingo-oophorectomy with no bowel resection provides very good results, and no clinical benefit has to date been clearly demonstrated when an excision of the infiltrating endometriosis is made. Therefore, it is essential that patients have accurate information regarding the benefits and risks associated with the procedure versus treatment without intestinal surgery.

In a paper recently submitted for publication, we reported the clinical results observed in 42 patients with DIE and rectovaginal or colorectal involvement who did not receive bowel surgery. According to our results, the patients who were treated only with hysterectomy and bilateral salpingo-oophorectomy demonstrated an excellent clinical evolution with no recurrences despite the long-term use of hormonal replacement therapy.

## 10. Relationship between Endometriosis and Ovarian Carcinoma

Another controversial aspect in endometriosis is its relationship with epithelial ovarian cancer. Sampson [90] was the first to describe this association, and his strict criteria have been used to identify malignant tumors that arise from endometriosis. Scott [91] also established that malignant transformation or transition occurred in benign ovarian endometriosis. A large number of publications have recently reported a clear pathogenesis of endometriosis-associated ovarian cancer, especially in the histological subtypes of clear cell carcinoma and endometrioid adenocarcinoma.

Atypical endometriosis has been described as a precursor lesion that can lead to certain types of ovarian cancer [92–95]. Moreover, endometriosis-induced inflammation and the auto- and paracrine production of sex steroid hormones could contribute to ovarian tumor genesis because these changes provide a microenvironment that favors the accumulation of sufficient genetic alterations and endometriosis-associated malignant transformations. These conditions induce the pathophysiological progression that begins with atypical epithelial proliferation (atypical endometriosis and metaplasia) followed by the formation of well-defined borderline tumors and finally culminating in full-blown malignant ovarian cancer [96].

Although several cases of endometriosis-associated ovarian carcinomas appear to be the final consequence of this pathological progression, others cases are not as obvious. Similar theories on the etiology, protective and risk factors, and common pathogenetic mechanisms have been postulated [97], but epidemiological findings regarding this association remain elusive.

Our group performed an observational study (currently submitted for publication) on 202 patients with epithelial ovarian tumors (EOT), and another set of 202 patients who had severe endometriosis. Our results showed that the patients with endometriosis were significantly younger than the patients with borderline or malignant tumors and that the patients with endometriosis-associated EOT and those patients with endometriosis-associated EOT were generally younger than the patients with malignant ovarian tumors without endometriosis. Moreover, most of the EOT patients were multiparous (63.9%), whereas most of the patients with endometriosis were nulliparous (65%). Regarding the tumor stage, 65% of the patients with malignant tumors were in stages III/IV, 47% of the patients with endometriosis-associated EOTs were in stage I, and 40% of the patients were in stages III/IV. In borderline tumors, endometriosis was associated in 10% of patients. In malignant tumors, endometriosis-associated endometrioid and clear cell histology was present in more than 40% of the patients, which contrasted with the lack of association with the other malignant histologies. Other interesting findings in this study were that endometrial carcinomas were observed in 5.4% of the patients with EOT, especially in the endometrioid and clear cell types. Breast cancer was diagnosed in 5.9% of the women with EOT compared with 0.5% diagnosed in the patients with endometriosis. In conclusion, we found a significant association between endometriosis (including atypical forms) and endometrioid and clear cell carcinomas but not with other EOT histological types. EOT were also associated with endometrial and breast carcinomas, particularly endometrioid and mucinous tumors, respectively.

The malignant transformation of endometriosis is a rare but reported complication of the disease. Ectopic endometrium promotes a local inflammatory response with activation of macrophages and cytokines that may have an overall negative effect on growth regulation, leading to premalignant changes either in the ectopic endometrium or in the implantation site itself. A transition of benign to atypical endometriosis is observed in 1%-2% of normal endometriotic tissues, whereas atypical endometriosis is considered to be precancerous and strongly associated with endometriosis-associated ovarian cancer. Epithelial ovarian cancers and adjacent endometriotic lesions have shown common genetic alterations, such as PTEN, p53, and bcl gene mutations, suggesting a possible malignant genetic transition spectrum [96].

Ovarian cancer can arise from the endometrioma, and it is possible to identify a transition spectrum from benign endometriosis to invasive disease within the same ovarian tissue, as shown by Wei et al. [96]. However, the most common finding of this procedure is an association between ovarian cancer and endometriosis in the same postmenopausal patient. One of our clinical cases is a 72-year-old woman who had a mixed serous endometrioid carcinoma on the left ovary with stromal luteinization (stage IIIc). She also had endometriosis on the right ovary and omentum, adenomyosis, leiomyomas, and endometrial hyperplasia. It is unknown whether the primary carcinoma was caused by preexisting endometriosis that developed into endometrioid carcinoma with stromal luteinization or whether the endometrioid carcinoma with a hormone-producing functional stroma reactivated a preexisting endometriosis. Wei et al. [96] and others support the first scenario, although the second one may also occur. Another one of our clinical cases showed a primary squamous cell carcinoma of the ovary, which was associated with endometriosis [98]. The relationship between endometriosis and ovarian carcinoma remains controversial.
